# Perceptions and Attitudes of Medical Students Toward the Integration of Large Language Models in Medical Education: Cross-Sectional Survey in China

**DOI:** 10.2196/66381

**Published:** 2026-03-09

**Authors:** Cheng Zhao, Weiqian Yan, Long Wang, Jing Wu, Herve Pasteur Ndikuriyo, Renhe Yu

**Affiliations:** 1 Department of Urology, Xiangya Hospital, Central South University, No.87 Xiangya Road, Kaifu District, Changsha, 410008, China, 86 13786116183, 86 89753011; 2 Department of Neurology, The Second Xiangya Hospital, Central South University, Changsha, China; 3 Department of Urology, The Third Xiangya Hospital, Central South University, Changsha, China; 4 Department of Education, Xiangya Hospital, Central South University, Changsha, China; 5 Department of Epidemiology and Health Statistics, Xiangya School of Public Health, Central South University, Changsha, China

**Keywords:** artificial intelligence, medical education, large language models, Chinese medical students, perceptions, attitudes, cross-sectional survey

## Abstract

**Background:**

Although artificial intelligence (AI) is being rapidly integrated into medical education, insights from medical students, particularly in the Chinese context, remain limited.

**Objective:**

This study was designed to explore Chinese medical students’ perceptions of and attitudes toward the integration of AI into medical education, as well as the factors that may influence their perspectives. The findings of this research offer valuable insights to assist medical educators in the effective implementation of these innovative educational approaches.

**Methods:**

On the basis of the estimated total number of clinical medical students at the target institutions, the sample size was calculated to be 379. A web-based questionnaire survey was designed to investigate the acceptance level of medical students toward the application of AI. The questionnaire consisted of 14 questions across 4 dimensions, which included demographic characteristics, perceptions of AI application, willingness, and concerns. Each dimension contained 3 to 4 questions. Descriptive statistics were used to tabulate the frequency of each variable. Chi-square tests and multiple regression analyses were conducted to measure the influencing factors.

**Results:**

A total of 566 cross-sectional online surveys were distributed from December 2023 to January 2024 through a snowball sampling method. Finally, 490 medical students from various local tertiary medical centers were involved. Overall, a majority of the participants showed a positive attitude toward future learning and the usage of AI, manifested as totally willing to acquire relevant knowledge (222/490, 45.3%), totally willing to use AI tools (230/490, 46.9%), and totally desiring that schools would offer AI-related courses (230/490, 46.9%). However, there is still a large proportion (392/490, 80.0%) of participants who expressed concerns regarding ethical issues. The findings also indicated that gender and educational level were significantly correlated with the AI application. Specifically, regression analysis indicated that male participants were more inclined to acquire AI information through social media (odds ratio 0.458, 95% CI 0.33‐0.67; *P*<.001) and that male or graduate-level participants were more likely to use AI for academic writing purposes (odds ratio 0.476, 95% CI 0.38‐0.82; *P*=.001 for male; odds ratio 1.552, 95% CI 1.32‐1.77; *P*=.009 for graduate students, respectively).

**Conclusions:**

Our findings indicate that a general awareness of AI’s role in medical education is evident among students. However, subgroup-specific differences must be taken into account, particularly when designing and optimizing educational strategies integrated with AI. This consideration is critical to ensuring that such tools align with the diverse learning needs of distinct student groups.

## Introduction

Artificial intelligence (AI) is increasingly used across various sectors, such as finance, health care, and education [1-4]. In the field of medical education, AI holds the potential to enhance teaching and learning by improving students’ critical thinking and communication skills [5]. However, while numerous studies have examined the views of medical educators, the perspectives of medical students themselves have received less attention. As modern medical education emphasizes collaboration between educators and learners, a thorough understanding of students’ attitudes is crucial for designing and implementing effective teaching strategies [6,7].

As China undergoes a broad transformation driven by AI, the distribution of educational resources in medicine remains uneven [8]. Currently, only a limited number of medical schools have established specialized AI courses, primarily located in economically developed regions [9]. This regional disparity in educational resources has led to widespread misconceptions about AI among medical students, which in turn may amplify ethical and privacy-related risks [10]. While Chinese policymakers acknowledge the need for regulation in this rapidly evolving field, most existing policies remain high-level guidelines, providing limited practical direction for specific sectors such as medical education [11].

To address the aforementioned issues, a cross-sectional study was conducted. The primary goal of this study was to (1) investigate the perceptions, attitudes, and willingness of medical students in China toward the integration of large language models (LLMs) into medical education; (2) identify key influencing factors associated with these perceptions and attitudes; and (3) provide empirical evidence to inform the design of targeted AI-related educational strategies for Chinese medical institutions and discuss the regional disparities while using this novel technology. No a priori hypotheses were formulated, given the exploratory nature of investigating region-specific perspectives on LLM integration in medical education.

## Methods

### Study Population

A web-based questionnaire survey was conducted in the central south region of China, and all participants were recruited from 2 regional tertiary hospitals in Changsha and 3 tertiary hospitals affiliated with Central South University. The inclusion criteria were as follows: (1) full-time students majoring in clinical medicine, (2) each participant could respond only once per account, (3) the total response time for completing the questionnaire was longer than 60 seconds, and (4) participants were recruited between December 2023 and January 2024. The exclusion criteria included (1) inability to complete the questionnaire in Chinese, (2) a history of cognitive impairment or mental illness, and (3) no prior clinical practice experience. Specifically, the assessment of cognitive impairment or mental illness was conducted via a self-reported question at the start of the questionnaire: “Have you ever been diagnosed with a cognitive disorder or mental illness by a professional physician?” Participants who answered “yes” were automatically excluded from the survey to ensure data quality and protect their well-being. This screening method was conducted in compliance with the Declaration of Helsinki [12]. Finally, we used the CHERRIES (Checklist for Reporting Results of Internet E-Surveys) to guide our report [13].

### Sample Size Calculation

The sample size for this cross-sectional survey was calculated to estimate the proportion of clinical medical students holding positive attitudes toward AI applications in medical education. The following formula for finite populations was used:

Formula: n=[N×z²×p(1−p)]/[(N−1)×e²+z²×p(1−p)]

Parameters: N=total population size (estimated at 2000 clinical students across target hospitals), z=1.96 (for a 95% confidence level), *P*=.50 (conservative proportion assuming maximum variability), and e=0.05 (desired margin of error)

Calculation:

n=[N×z²×p(1-p)]/[(N-1)×e²+z²×p(1-p)]

=[2000×(1.96)²×0.5×.5]/[(1999×0.0025)+(3.8416×0.25)]

=[2000×3.8416×.25]/[4.9975+0.9604]

=1920.8/5.9579 ≈ 322

Nonresponse adjustment (15% attrition):

n_adjusted=322/0.85 ≈ 379

A minimum of 379 participants was required.

### Questionnaire Design

The questionnaire was developed based on a comprehensive review of the existing literature on related topics and was modified according to local perceptions in China [14,15]. As most of the participants included in our study were preclinical medical students, the questionnaire items were designed to avoid esoteric or ambiguous research-related or clinical questions as much as possible. Before the formal investigation, a pilot test of the questionnaires was conducted in this study. Three experts (LW, JW, and WY) were invited to conduct a systematic content validity evaluation. Subsequently, 20 students were recruited from different clinical disciplines, maintaining a male-to-female ratio of 1:1. The participants included both undergraduate and postgraduate students. These medical students completed the questionnaire to identify any encountered issues. On the basis of their feedback, the questionnaire was further refined. It should be noted that the responses from the pilot test were excluded from the final data analysis. The final questionnaire, consisting of 14 questions, was published on the WJX platform [16]. It addressed four dimensions: demographic characteristics, perceptions, willingness, and concerns. Each dimension encompassed 3 to 4 questions, and depending on the research target, question formats were either single or multiple choices.

### Distribution of Questionnaire

We distributed the online questionnaire through “WeChat,” (Tencent Holdings Limited) a widely used real-time messaging application in China. The team members contacted educators from various hospitals through WeChat, informed them of the purpose of the survey, and then forwarded the questionnaire to them in WeChat groups of students from different grades. Medical students who agreed to participate scanned the QR code to anonymously answer the questionnaire online. To further enhance the reach and minimize potential bias, we used a snowball sampling method by requesting initial respondents to forward the survey link to other medical students in their networks [17].

### Ethical Considerations

Informed consent and instructions were displayed at specific locations in the questionnaire. Participants were required to complete an informed consent form, which stated, “You acknowledge and agree that the data collected may be used for research purpose,” as a prerequisite for proceeding with the survey. Participants could freely modify their selected options before final submission and retract their questionnaire at any time after delivery.

Given the nature of the study (anonymous, noninterventional, and without collection of personal or sensitive data), it was exempt from formal institutional review board review and approval, in accordance with the ethical guidelines of the Medical Ethics Committee of Xiangya Hospital, Central South University. The study was conducted in full compliance with relevant ethical principles, ensuring the protection of participants’ privacy and rights throughout the research process. As the survey was voluntary and designed to take no more than 10 minutes to complete, no financial or nonfinancial compensation was provided to participants.

### Data Analysis

After questionnaire collection, data cleaning was conducted. The questionnaires were screened based on the following predetermined criteria: (1) responses with excessive missing data, accounting for two-thirds of the total questions; (2) responses exhibiting uniform or highly regular answer selections; and (3) questionnaires completed in less than 60 seconds. These time thresholds were established as reasonable response times based on the pilot test conducted previously.

We used SPSS Statistics (version 26; IBM Corporation) for data analysis. First, descriptive analysis was performed to summarize the frequency distribution characteristics of the variables. Subsequently, subgroup analysis using chi-square tests was conducted to explore the associations between different demographic characteristics. Finally, regression analysis was applied to control for confounding factors and further investigate the associations between factors such as gender, age, and education level with the study objectives. If *P*<.05, differences were considered statistically significant. The reliability of the items from the willingness and concerns dimensions was tested using the Cronbach α coefficient analysis. The results of the Cronbach α were considered to have an acceptable reliability, as the generally accepted rule is that α values of 0.6 to 0.7 indicate an acceptable level of reliability, and 0.8 or greater is a very good level [18].

## Results

### Demographic Characteristics

A total of 566 questionnaires were distributed online, and 490 valid responses were collected (a response rate of 86.6%). The demographic characteristics of the participants are presented in Table 1. Overall, the gender distribution of participants was balanced (248/490, 50.7%, men vs 242/490, 49.3%, women), with the majority concentrated in the age range of 20 to 25 years (304/490, 62.0%), and only 32 individuals aged 35 years and older (32/490, 6.5%). The majority of participants were undergraduate students (258/490, 52.7%), followed by master’s candidates (134/490, 27.3%), PhD candidates (74/490, 15.1%), and postdoctoral fellows (16/490, 3.3%). To clarify the Chinese medical education model, the following definitions apply to participant degree programs: (1) undergraduate students, that is, students pursuing a 5-year Bachelor of Medicine degree with a primary focus on foundational medical courses and who are not concurrently enrolled in other degree programs; (2) graduate students, including master’s (3-y program) and PhD (3‐ to 4-y program) students, who specialize in clinical subfields or medical research and are required to complete clinical rotations and publish academic papers; and (3) postdoctoral fellows, who are researchers who have completed a PhD and engage in advanced clinical or research work at hospitals; while not “students” in the traditional sense, they were included because they continue to participate in medical education training programs and represent the upper end of the medical learner spectrum. This classification aligns with national medical education standards in China [19].

**Table 1.** Demographic characteristics of study (N=490).

Characteristics	Values (%)
Sex	
Male	248 (50.7)
Female	242 (49.3)
Age (y)	
<20	8 (1.6)
21‐25	304 (62.0)
26‐30	100 (20.4)
31‐35	46 (9.4)
>35	32 (6.5)
Educational level	
Junior college	8 (1.6)
Undergraduate degree	258 (52.7)
Master’s degree	134 (27.3)
PhD	74 (15.1)
Postdoctorate	16 (3.3)

The specific frequency distribution is as follows: The age range of participants is from 18 to 38 years, and the median age is 23.1 (18.7-31.6) years. The extreme values were defined as ages less than 20 (8/490, 1.6%) or greater than 35 (32/490, 6.5%) years. Specifically, the proportion of participants aged less than 20 years was (8/490, 1.6%), all of whom were undergraduate students in their first or second year. Most participants (304/490, 62.0%) were aged 21 to 25 years, corresponding to undergraduate fourth- or fifth-year students or first-year postgraduates, followed by those aged 26 to 30 years (100/490, 20.4%), who were second- or third-year postgraduates, and those aged older than 30 years (78/490, 15.9%), who were PhD or postdoctoral fellows. This distribution is consistent with the age structure of medical students in China, where most enter postgraduate studies after 23 years of age [19].

### Perceptions of AI Application

This set of questions primarily focused on participants’ perceptions of the application of LLMs. The description of participants’ perceptions of LLMs is presented in Tables2^4^. Subsequently, we conducted chi-square tests to investigate the correlation between other factors and perceptions. First, male participants were more likely to have been exposed to information related to LLMs before the conduction of the survey (158/248, 63.7% vs 132/242, 54.5% for women; *χ*²_1_=5.4; *P*=.02), as well as educational level also contributed to this issue (undergraduates: 162/258, 62.7%; master’s students: 80/134, 59.7%; PhD candidates: 30/74, 40.5%; *χ*²_1_=19.854; *P*=.001). Second, gender (180/248, 72.5% vs 130/242, 53.7% for women; *χ*²_1_=18.7; *P*<.001), age (21‐25 y: 142/304, 46.7%; 26‐30 y: 60/100, 60%; and 31‐35 y: 38/46, 82%; *χ*²_1_=26.4; *P*<.001), and educational level (undergraduates: 170/258, 65.8%; master’s students: 68/134, 50.7%; PhD candidates: 56/74, 75.6%; *χ*²_1_=38.7; *P*<.001) were all correlated with the way that participants primarily acquired LLM-related information. A clear trend was observed that as education level increased, participants were more likely to access LLM information via academic journals or friends chatting and to recognize the value of LLM for research. Third, gender (206/248, 83.0% vs 168/242, 69.4% for women; *χ*²_1_=12.6; *P*<.001), age (21‐25 y: 222/304, 73.0%; 26‐30 y: 84/100, 84.0%; 31‐35 y: 40/46, 87.0%; *χ*²_1_=25.5; *P*<.001), and educational level (undergraduates: 186/258, 72.1%; master’s students: 110/134, 82.1%; PhD candidates: 60/74, 81.1%; *χ*²_1_=22.5; *P*<.001) also influenced participants’ views on the application methods of LLMs in the medical field, especially in assisting in academic writing and literature translation. Finally, age (21‐25 y: 232/304, 76.3%; 26‐30 y: 84/100, 84%; 31‐35 y: 42/46, 91.3%; *χ*²_1_=25.7; *P*<.001) and educational level (undergraduates: 198/258, 76.7%; master’s students: 110/134, 82.1%; PhD candidates: 70/74, 94.6%; *χ*²_1_=25.0: *P*<.001) were also correlated with the challenges of LLMs in medical applications, specifically concerns about privacy protection. These trends indicated that older participants expressed greater concern about privacy risks compared to younger participants, which may reflect 2 key aspects: first, a higher demand for AI applications in the academic domain among more experienced learners, and second, a stronger awareness of data protection among this group.

**Table 2.** Chi-squared test between gender and categorical variables.

Variable	Gender, n		Pearson *χ*² (*df*)	*P* value^a^
	Male	Female		
Have you ever been exposed to information related to large language models?			5.4 (1)	.02
Yes	158	132		
No	90	110		
In what ways have you primarily acquired information about large language models?			18.7 (1)	<.001
Social media				
Yes	180	130		
No	68	112		
Friend chatting				
Yes	118	138		
No	130	104		
What do you think large language models can be used for in the field of medicine?			12.6 (1)	<.001
Assistance in writing academic papers				
Yes	206	168		
No	42	74		
Translation of literature				
Yes	186	160		
No	62	82		

a *P*<.05 was considered statistically significant.

**Table 3.** Chi-squared test between age and categorical variables.

Variable	Age (y), n					Pearson *χ*² (*df*)	*P* value^a^
	<20	21‐25	26‐30	31‐35	>35		
In what ways have you primarily acquired information about large language models?						26.4 (1)	<.001
Friend chatting							
Yes	2	142	60	38	14		
No	6	162	40	8	18		
What do you think large language models can be used for in the field of medicine?						25.5 (1)	<.001
Assistance in writing academic papers							
Yes	2	222	84	40	26		
No	6	82	16	6	6		
Translation of literature							
Yes	0	208	80	36	22		
No	8	96	20	10	10		
What challenges do you believe large language models encounter in their applications within the field of medicine?						25.7 (1)	<.001
Privacy protection							
Yes	2	232	84	42	30		
No	6	72	16	4	2		

a *P*<.05 was considered statistically significant.

**Table 4.** Chi-squared test between education level and categorical variables.

Variable	Education level, n					Pearson *χ*² (*df*)	*P* value^a^
	Junior college	Undergraduate degree	Master’s degree	PhD	Postdoctorate		
Have you ever been exposed to information related to large language models?						19.9 (1)	.001
Yes	6	162	80	30	4		
No	2	96	54	44	12		
In what ways have you primarily acquired information about large language models?						38.7 (1)	<.001
Academic journal							
Yes	2	102	64	36	12		
No	6	156	70	38	4		
Social media							
Yes	4	170	68	56	12		
No	4	88	66	18	4		
Lectures							
Yes	0	86	18	14	6		
No	8	172	116	60	10		
Friend chatting							
Yes	2	108	94	38	14		
No	6	150	40	36	2		
What do you think large language models can be used for in the field of medicine?						22.6 (1)	<.001
Summarize medical research papers							
Yes	6	216	124	72	14		
No	2	42	10	2	2		
Assistance in writing academic papers							
Yes	2	186	110	60	16		
No	6	72	24	14	0		
Translation of literature							
Yes	2	172	102	56	14		
No	6	86	32	18	2		
What challenges do you believe large language models encounter in their applications within the field of medicine?						25.0 (1)	<.001
Privacy protection							
Yes	4	198	110	70	8		
No	4	60	24	4	8		

a *P*<.05 was considered statistically significant.

### Willingness

**Figure 1.**
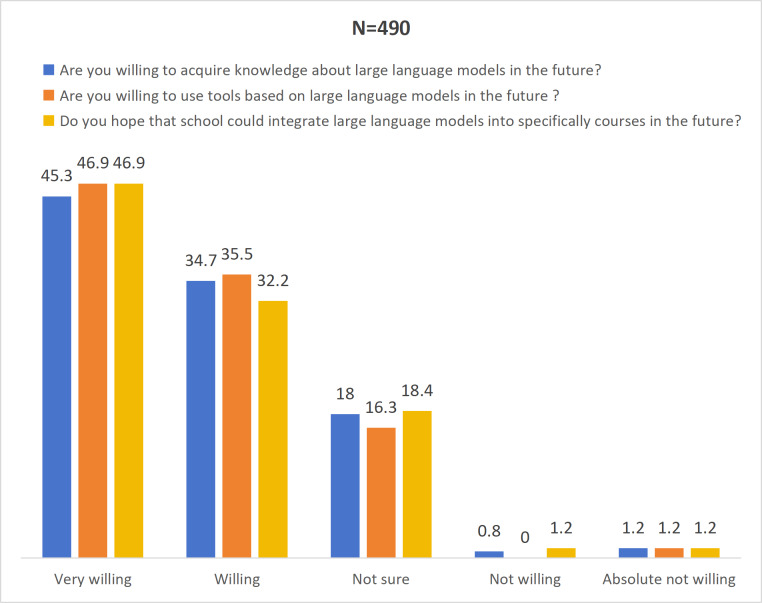
Descriptive statistical analysis of the willingness domain. Numbers represent the percentage of participants in each category.

The 3 questions in this dimension examined the participants’ attitudes regarding their future studies and the application of or training courses related to LLMs. The responses were recorded using a 5-point Likert scale, ranging from 1 (totally agree) to 5 (totally disagree). Overall, all the participants demonstrated a positive willingness to accept and engage with AI-related learning and training. Specifically, the proportion of participants who chose “completely agree” accounted for nearly half of the total number in all 3 questions. Additionally, the median scores were all relatively low (with lower scores indicating a higher degree of acceptance by the participants). Specific data are present in Figure 1.

### Concerns

**Figure 2.**
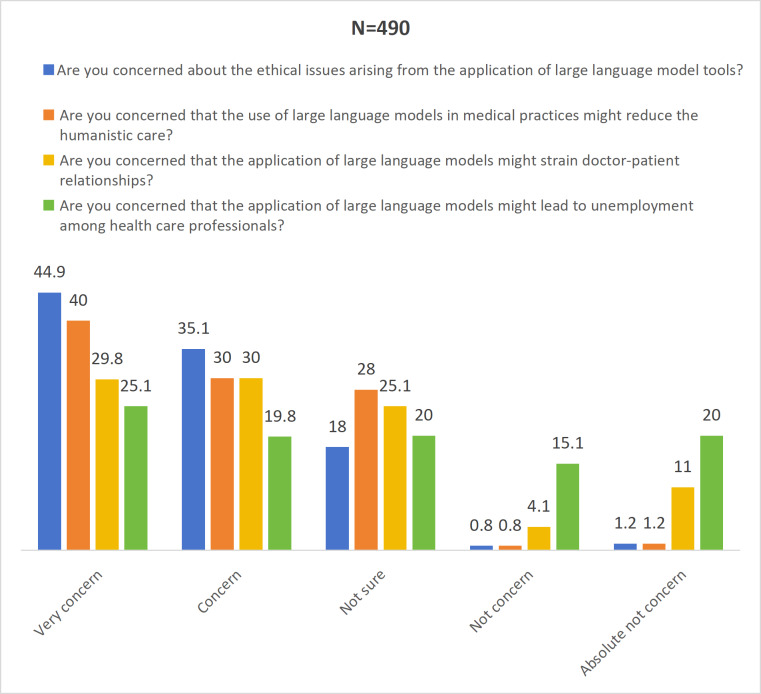
Descriptive statistical analysis of the concerns domain. Numbers represent the percentage of participants in each category.

We also assessed potential concerns about the integration of LLMs into medical education. First, the majority of participants expressed concerns about ethical issues, with more than half of them choosing to totally agree. However, in the subsequent questions related to medical issues, the number of participants choosing to totally agree largely decreased compared to the ethical questions. Moreover, regarding the issue of whether LLMs would lead to the unemployment of physicians, the vast majority of participants did not express any significant concern, with the median scores being relatively high. Specific data are shown in Figure 2.

### Regression Analysis

After adjusting for gender, age, and education, we observed that both gender and educational level had a significant influence on participants’ awareness and attitudes. Specifically, male participants were more inclined to acquire relevant information through social media (odds ratio [OR] 0.458, 95% CI 0.33‐0.67; *P*<.001). Furthermore, after adjusting for other variables, male participants (OR 0.476, 95% CI 0.38‐0.82; *P*=.001) and graduate students (OR 1.552, 95% CI 1.32‐1.77; *P*=.009) both showed a strong inclination toward using AI for academic writing. Data are presented in Table 5.

**Table 5.** Binary logistic regression between the categorical variables and different options.

Characteristics	Social media			Assistance in writing academic papers		
	*P* value	OR^a^ (95% CI)		*P* value	OR (95% CI)	
Sex	<.001^b^			.001^b^		
Male		Ref.^c^			Ref.	
Female		0.458 (0.33‐0.67)			0.476 (0.38‐0.82)	
Education level	.81			.009^b^		
Undergraduate degree		Ref.			Ref.	
Graduate degree		1.033 (0.95‐1.21)			1.552 (1.32‐1.77)	

a OR: odds ratio.

b *P*<.05 was considered statistically significant.

c Ref.: reference.

## Discussion

### Principal Findings

To summarize the principal findings of this study: Among 490 valid responses from Chinese medical students in Hunan, (1) participants generally held a positive attitude toward LLM integration into medical education, with 45.3% expressing full agreement with learning AI-related knowledge and 46.9% willing to use AI tools; (2) gender and educational level significantly influenced LLM-related perceptions—men were more likely to access LLM information via social media and use LLMs for academic writing, while postgraduates compared with undergraduates showed stronger recognition of the LLM’s academic value; (3) ethical concerns were the most prominent worry, followed by privacy risks, but few participants feared LLM-induced physician unemployment; and (4) most students acquired LLM information through informal channels (social media or friends chatting), with only a few relying on formal medical school courses (academic journals or lectures).

### Implications of Findings

Importantly, this study does not claim that students possess “substantial foundational knowledge of AI”—instead, it reports students’ self-reported awareness and use of AI. No conclusions can be drawn about students’ actual mastery of AI foundational knowledge because of the self-reported data limitations. However, for AI course design, the study’s findings highlight specific gaps rather than definitive knowledge levels: (1) informal information channels, such as social media, may lead to an incomplete understanding of AI principles—thus, courses should include foundational modules on AI basics; (2) high ethical concerns suggest a need for modules on AI ethics (eg, case studies of AI-related academic misconduct and guidelines for patient data protection); and (3) the preference for research applications indicates that courses should integrate AI tools for thesis writing, literature review, and data analysis (aligned with Chinese medical education requirements). Future research should use objective assessments to identify specific knowledge gaps and inform the targeted curriculum design.

### Comparison to the Literature

This study’s findings align with some conventional knowledge in medical education while revealing region-specific differences.

Findings aligning with existing literature include the following. First, informal AI learning channels: consistent with one study [20], our results show that most Chinese medical students (63.2% and 52.2%) access AI information via nonformal channels (social media or friends chatting) rather than school courses, which aligns with global trends [20,21]. Specifically, 1 study has demonstrated that, on the premise of enhancing transparency and interpretability, leveraging social media as a channel helps expand the audience of medical research and provides opportunities for the early intervention of diseases [22]. However, another study also indicates that social media are associated with risks of data misuse, and its fundamental flaw lies in the lack of procedural standards and extremely low transparency. As an emerging field, AI will further amplify the risks when students acquire relevant information through social media platforms [23]. Second, gender differences in AI attitudes: our findings indicated that male participants were more likely to use AI (68.2% vs 51.2% of female participants), whereas female participants expressed greater concerns about risks (72.3% vs 54.8% of male participants). This result is consistent with surveys from other studies [24-26], which suggest cross-cultural consistency in gender-related AI perceptions. Third, low formal AI education coverage: our data (25.3% of students with formal AI courses) align with a Chinese survey reporting that regional disparities of AI-related instruction exist among medical schools in China, confirming the existence of a national education gap [9].

Novel or region-specific findings are as follows. First, preference for research over clinical AI use: unlike Western studies [27-29], where medical students prioritize AI for clinical tasks, our participants favored research applications (76.3% vs 32.7% for clinical use). This difference could be explained by China’s medical education requirements, which mandate research experience and paper publication for postgraduates [19]. Second, low fear of AI-induced unemployment: only 12.7% of our participants worried about physician unemployment due to AI. This may reflect China’s large health care workforce gap [30], where AI is viewed as a complement rather than a replacement for physicians. Notably, all participants in our study were recruited from large tertiary hospitals located in provincial capitals; therefore, this conclusion may be subject to selection bias. Third, high ethical concerns focused on academic misconduct: while Western studies highlight clinical ethics [31,32], our participants prioritized academic ethics more likely due to strict academic integrity policies in Chinese medical schools [33].

At the policy level, the value of policy guidance in the field of AI applications has gained growing recognition. These policy frameworks not only establish effective norms for AI implementation in practical scenarios but also play a pivotal role in mitigating risks associated with AI deployment [34]. China’s regulatory environment is characterized by centralized management and alignment with national strategic priorities, emphasizing that technological development should serve the overall interests of society, safeguard national security, and uphold social stability and moral norms [11]. This top-down approach facilitates coordinated deployment of AI in health care and education, ensuring consistency with long-term national goals. For example, the “Implementation Opinions on Promoting and Regulating the Application and Development of “AI+Healthcare” (issued by the National Health Commission and 4 other ministries in November 2025) sets targets for 2027 and 2030, explicitly supporting AI application in clinical scenarios [35]. Moreover, the “Reference Guidelines for AI Application Scenarios in the Health Industry” (issued by the National Health Commission in November 2024) defines 84 AI application directions in health care, including medical image analysis and intelligent drug research—providing clear guidance for designing AI education modules aligned with clinical needs [36]. These policies’ emphasis on “application-oriented AI development” aligns with our finding that medical students prioritize AI use for academic and clinical tasks, highlighting the need for education to bridge the gap between policy goals and student practice. In contrast, the regulatory environment in Western countries adopts a more decentralized and issue-specific focus. For example, Europe prioritizes robust protection of personal privacy and data sovereignty, as exemplified by the General Data Protection Regulation and the EU AI Act [37], which classifies medical AI as “high risk” and mandates strict transparency and safety requirements. The United States emphasizes fostering technological innovation and market competition, often relying on industry self-regulation supplemented by targeted federal guidelines [38]. These measures exemplify China’s proactive efforts toward digital transformation in the health care and education sectors, with policy acting as a catalyst for cross-sector collaboration.

Notably, disparities in resource allocation between different regions may give rise to structural differences in the AI literacy levels of medical students [39]. This gap is unlikely to be alleviated in the short term. In Hunan Province, as a relatively underdeveloped province located in the central-southern area of China, there are relatively fewer high-quality AI medical resources compared to economically developed coastal provinces. Although the “Artificial Intelligence+Teacher Team Construction Project” initiated by Hunan Province may promote the balanced development of education within the province [40], this policy is unlikely to be promoted to other provinces. Moreover, the concept that this policy views AI as a tool rather than a decision-making entity may inhibit students’ critical thinking about the ethical risks of AI, thereby increasing the potential risks of its usage. Therefore, as the primary cohort of future users of AI-related applications, their insights are critical to facilitating the effective and contextually relevant adoption of this transformative technology, particularly in aligning its use with the unique demands of medical practice and education. These findings are not only regionally relevant but also have a significant impact on the digital transformation of medical education nationwide. However, when applying these findings to other regions, adjustments must be made based on the differences in the local policy and medical ecosystems.

### Limitations

Notably, this study has several limitations. First, cross-sectional studies, although useful for identifying correlations between variables such as the use of LLMs and learning outcomes, are limited in their ability to establish causality. Second, while the sample is representative of the targeted region, its regional specificity may preclude full generalization to the broader population of Chinese medical students. Third, the relatively small sample size of the questionnaire survey may render certain conclusions vulnerable to random variation; expanding the sample size could potentially alter the robustness of these results. Furthermore, selection bias may exist, as medical students with limited interest in the medical applications of LLMs and those who are currently experiencing emotional distress were likely less motivated to participate in the survey. This nonresponse bias may have led to an overrepresentation of positive attitudes toward the use of LLMs in medical contexts. Finally, the study lacks data from two key stakeholders in the medical education ecosystem: practicing physicians and patients. Their perspectives on AI applications in medical practice and education would serve as a valuable complement to the current findings.

### Conclusions

In summary, while the momentum toward AI integration in medicine is undeniable, its healthy development relies on coordinated efforts to advance technology responsibly, implement proactive regulation, and prioritize the diverse perspectives of those most impacted by its implementation. This approach will not only address the limitations identified in this study but also lay the groundwork for an AI-enabled medical ecosystem that is equitable, safe, and aligned with the core goals of health care and medical education.

## Abbreviations

AI: artificial intelligence
CHERRIES: Checklist for Reporting Results of Internet E-Surveys
LLM: large language model
OR: odds ratio
